# Geographic bias related to geocoding in epidemiologic studies

**DOI:** 10.1186/1476-072X-4-29

**Published:** 2005-11-10

**Authors:** M Norman Oliver, Kevin A Matthews, Mir Siadaty, Fern R Hauck, Linda W Pickle

**Affiliations:** 1 Department of Family Medicine, University of Virginia, Charlottesville, VA, USA; 2 Department of Public Health Sciences, University of Virginia, Charlottesville, VA, USA; 3 Surveillance Research Program, Division of Cancer Control and Population Sciences, National Cancer Institute, Bethesda, MD, USA

**Keywords:** bias (epidemiology), confounding factors, epidemiology, geographic information systems

## Abstract

**Background:**

This article describes geographic bias in GIS analyses with unrepresentative data owing to missing geocodes, using as an example a spatial analysis of prostate cancer incidence among whites and African Americans in Virginia, 1990–1999. Statistical tests for clustering were performed and such clusters mapped. The patterns of missing census tract identifiers for the cases were examined by generalized linear regression models.

**Results:**

The county of residency for all cases was known, and 26,338 (74%) of these cases were geocoded successfully to census tracts. Cluster maps showed patterns that appeared markedly different, depending upon whether one used all cases or those geocoded to the census tract. Multivariate regression analysis showed that, in the most rural counties (where the missing data were concentrated), the percent of a county's population over age 64 and with less than a high school education were both independently associated with a higher percent of missing geocodes.

**Conclusion:**

We found statistically significant pattern differences resulting from spatially non-random differences in geocoding completeness across Virginia. Appropriate interpretation of maps, therefore, requires an understanding of this phenomenon, which we call "cartographic confounding."

## Background

Epidemiologists and public health researchers are increasingly using geographic information systems (GIS) to assess the association between population health and the area characteristics of where people reside [1-8]. However, spatial analyses are fraught with challenges. The initial task of assigning geographic locations to study subjects – geocoding – can be difficult [9,10]. As Krieger et al have noted [11], the completeness with which geocoding is performed varies, which can affect the findings of spatial epidemiologic analyses [12].

There are two separate and equally important problems that can arise in the process of matching addresses to locations. First, addresses may be assigned longitudes and latitudes that are unacceptably far from their actual locations. This positional inaccuracy, owing to incorrect addresses or the assignment of incorrect latitudes and longitudes to correctly recorded addresses, can lead to bias in a study's outcomes [13-16]. This paper does not address this issue; rather, it focuses on the second problem, which is differential match rates by geographic region.

Such differential match rates can give biased results because GIS analyses may be based on unrepresentative data and a consequent information [17] bias in which important data are missing in a non-random fashion. Non-random missingness, a term used by statisticians to describe this information bias [18], can result from social, economic, political, and other reasons. One of the central contributions of spatial analyses of disease is that geographic location can be used to help account for unmeasured and unmeasurable risks for disease[19,20]. However, if the risk factors for missing locations are some of the same risks as those for the disease under study, then this confounding of risk factor with place makes an unbiased spatial analysis more difficult to achieve [21]. We illustrate this latter type of bias through an analysis of prostate cancer incidence over a 10-year period in Virginia.

In a study of prostate cancer incidence and race in Virginia [22], we found that the median household income and urban status of an area were associated positively with prostate cancer incidence for both African Americans and whites. The level of poverty and lower education were associated with decreased incidence among whites but not African Americans. Statistically significant associations were found only at the census tract level, disappearing when the analyses were conducted at the county level.

We sought to discern whether the differences we noted at the census-tract level analysis were real or simply an example of the modifiable areal unit problem (MAUP) [20], in which one obtains different results and inferences when the same set of data is grouped in different sized areal units. For example, Krieger et al [4] found in their analysis of cancer incidence and mortality in Massachusetts and Rhode Island that significant findings were lost when the analyses were conducted with areal units larger than the census tract. Gregorio et al [23], however, found in their cluster analysis of incident prostate and breast cancer cases in Connecticut that there were few differences in results across areal units, and there was no compelling need in typical cancer surveillance studies to prepare data at areas finer than the census tract.

In our study, only 74% of the cases were geocoded to the census-tract level, whereas 100% of the cases had county codes. When the statistical analyses were conducted at the county level with the same reduced data set (74% of the cases) as that used at the census-tract level, the results were similar, regardless of the geographic unit of analysis. (The generalized linear mixed modeling used to conduct this analysis and the results are described in detail elsewhere [22].) When the county analyses were conducted using 100% of the cases, the associations between predictor variables and prostate cancer incidence disappeared. These statistical analyses indicated the differences in results were not owing to the MAUP, but possibly to missing data.

In this paper, we report the findings of a study we conducted to evaluate whether unrepresentative data resulted merely from being missing, or whether the "missingness" of the data itself was confounded geographically with our covariates.

## Results

County codes were available for all African-American and white cases from the Virginia Cancer Registry (VCR). We successfully geocoded 26,338 (74 percent) of the cases to the census-tract level. The types of unmatched cases did not differ between African Americans and whites, with rural routes and Post Office boxes making up virtually all of the 26 percent of unmatched addresses in both populations. (See Table 1.) As can be seen from Table 1, we geocoded about 94% of the cases that possibly could be geocoded. Gregorio et al [21] note that subject loss between 5 and 16% had been reported in a few studies [24-26]. Our geocoding match rate falls within that same range when measured against the cases with actual addresses.

**Table 1:** Census Tract geocoding results broken down by address type.

			**% Of address types (No.)**			
			
	**No.**	**%(No.)**	**Street Addresses**	**Rural Routes^a^**	**P.O. Boxes^a^**	***Other^a,b^***
Matched^c^						
African American	6,060	74.0	93.8	0.0	0.0	0.0
White	20,278	73.4	94.0	0.0	0.0	0.0
						
Unmatched^c^						
African American	2,192	26.6	6.2	100.0	100.0	100.0
White	7,136	26.0	6.0	100.0	100.0	100.0
TOTAL NUMBER	35,666	(26,338)	(28,039)	(4,131)	(2,635)	(861)

^a^Accurate geocoding to the Census tract cannot be performed on this address type. ^b^Includes garbled and incomplete addresses. ^c^To the Census Tract.

**Figure 1:**
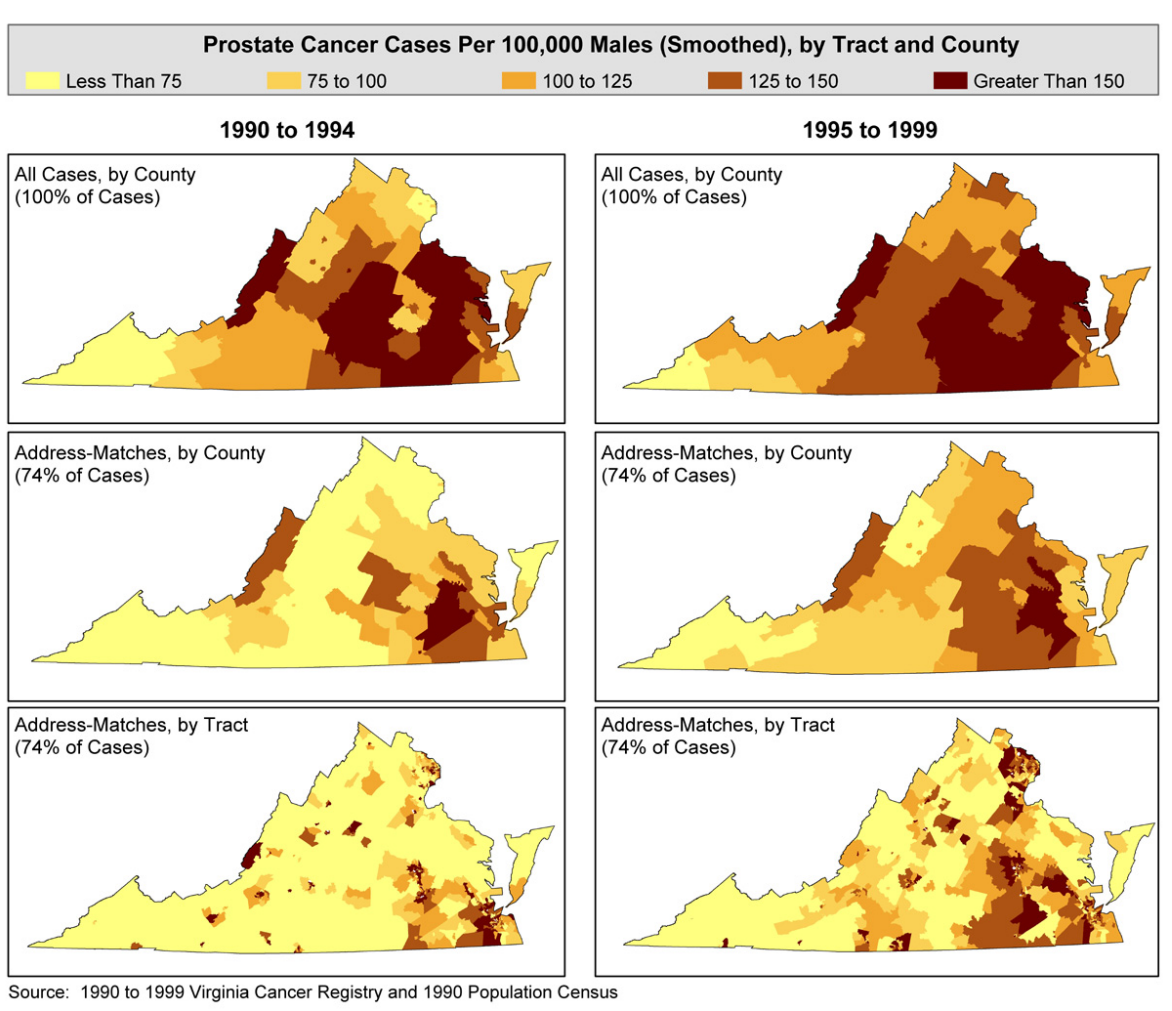
Annualized, age-adjusted prostate cancer incidence in Virginia, 1990–99.

**Figure 2:**
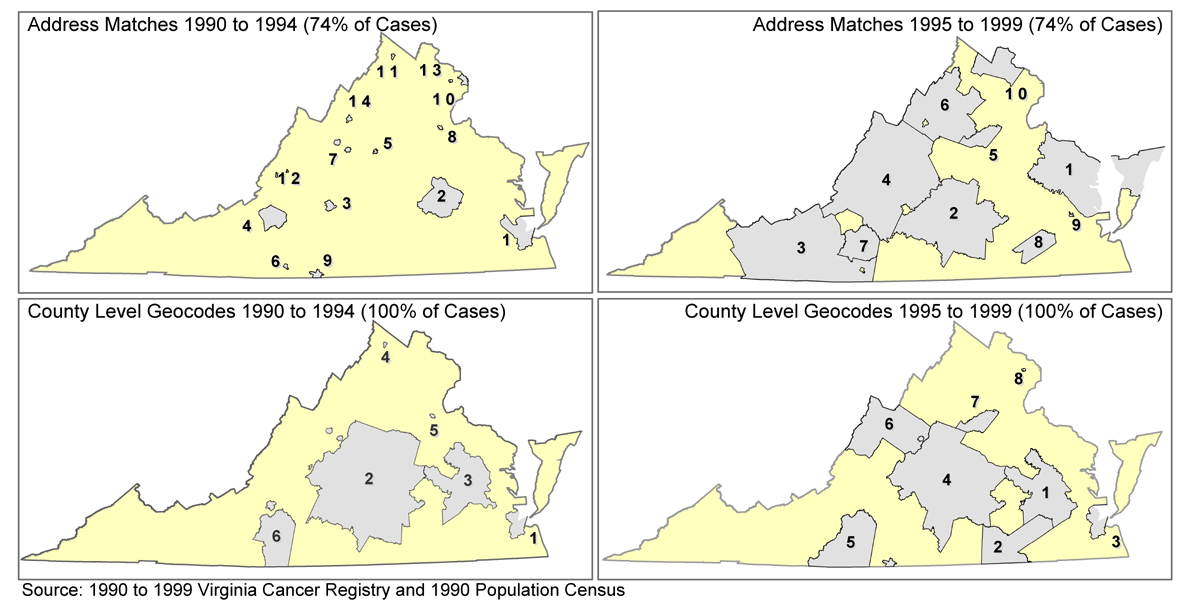
Prostate cancer incidence clusters in Virginia, 1990–99.

The overall incidence rate for whites was 97/100,000, whereas the rate for African Americans was 157/100,000, using the cases successfully geocoded to the census tract. (Smoothed maps of the annualized, age-adjusted prostate cancer rates for all males in Virginia 1990–99 are shown in Figure 1.) Statistical testing for global clustering was highly significant for the entire time period (Tango's Maximum Excess Events Test, p < 0.008) and for 1990–94 and 1995–99 separately (p < 0.001 for both). We examined local clustering at the county level in both time periods, using either 100 percent of the cases or just those cases geocoded to the census tract level (74 percent of the cases). With 100 percent of the cases, we found 6 such clusters in 1990–94 and 8 such clusters in 1995–99. In both time periods, major clusters appeared in geographically similar locations. For the reduced data set, we found 14 statistically significant clusters in 1990–94 and 10 such clusters in 1995–99. For each time period, patterns appeared markedly different, depending upon whether one used the cases located in the county or those geocoded to the census tract (Figure 2) [22].

**Figure 3:**
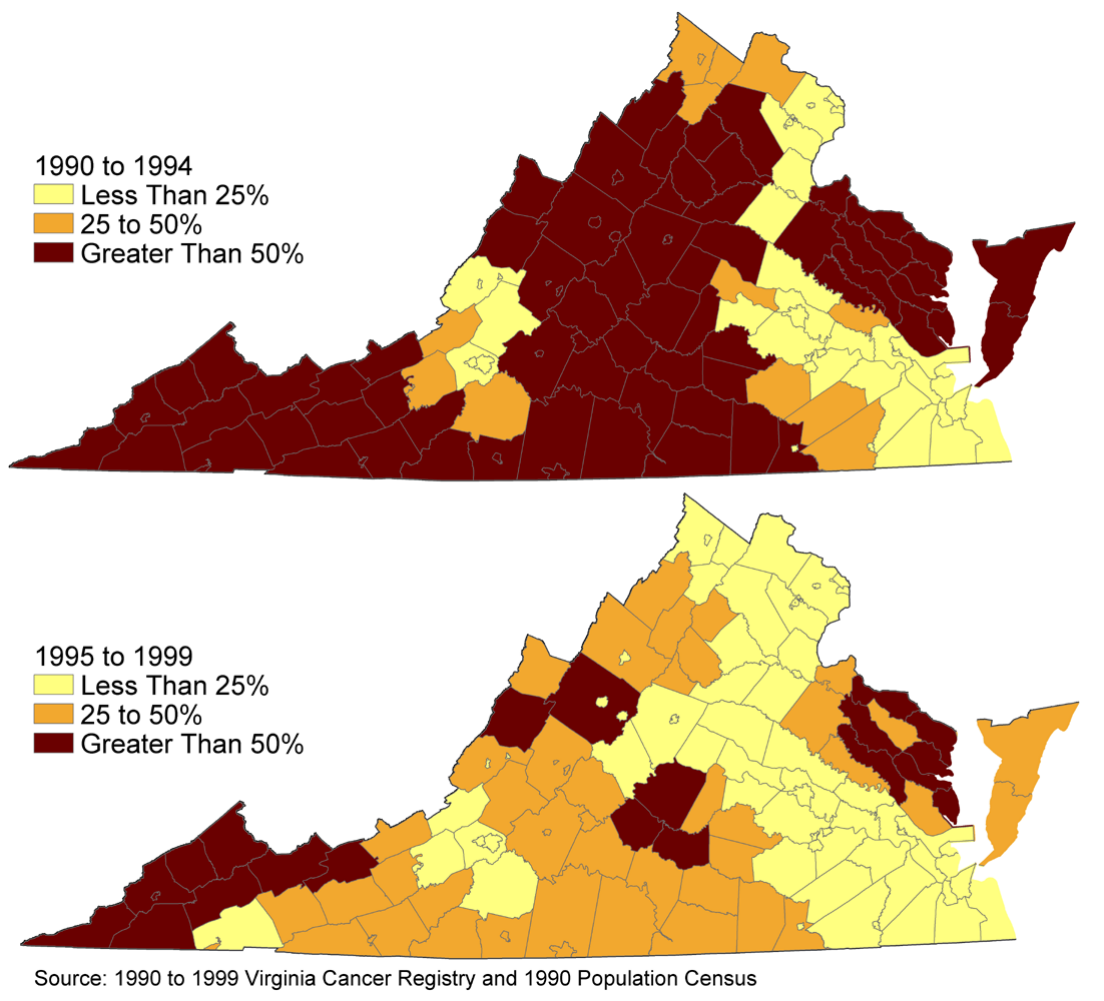
Proportion of unmatched prostate cancer cases in Virginia, 1990–99.

Figure 3 shows the proportion of missing census tract geocodes for the years 1990–1994 and 1995–1999. During the earlier study period, the proportion of missing geocodes in some areas reached 95%. Clearly, in the second half of the study period, we successfully geocoded more cases. In part, the increased match rate was due to a decreasing rural population, and, also, owing to increased numbers of rural residents receiving addresses rather than rural routes or Post Office box numbers.

**Figure 4:**
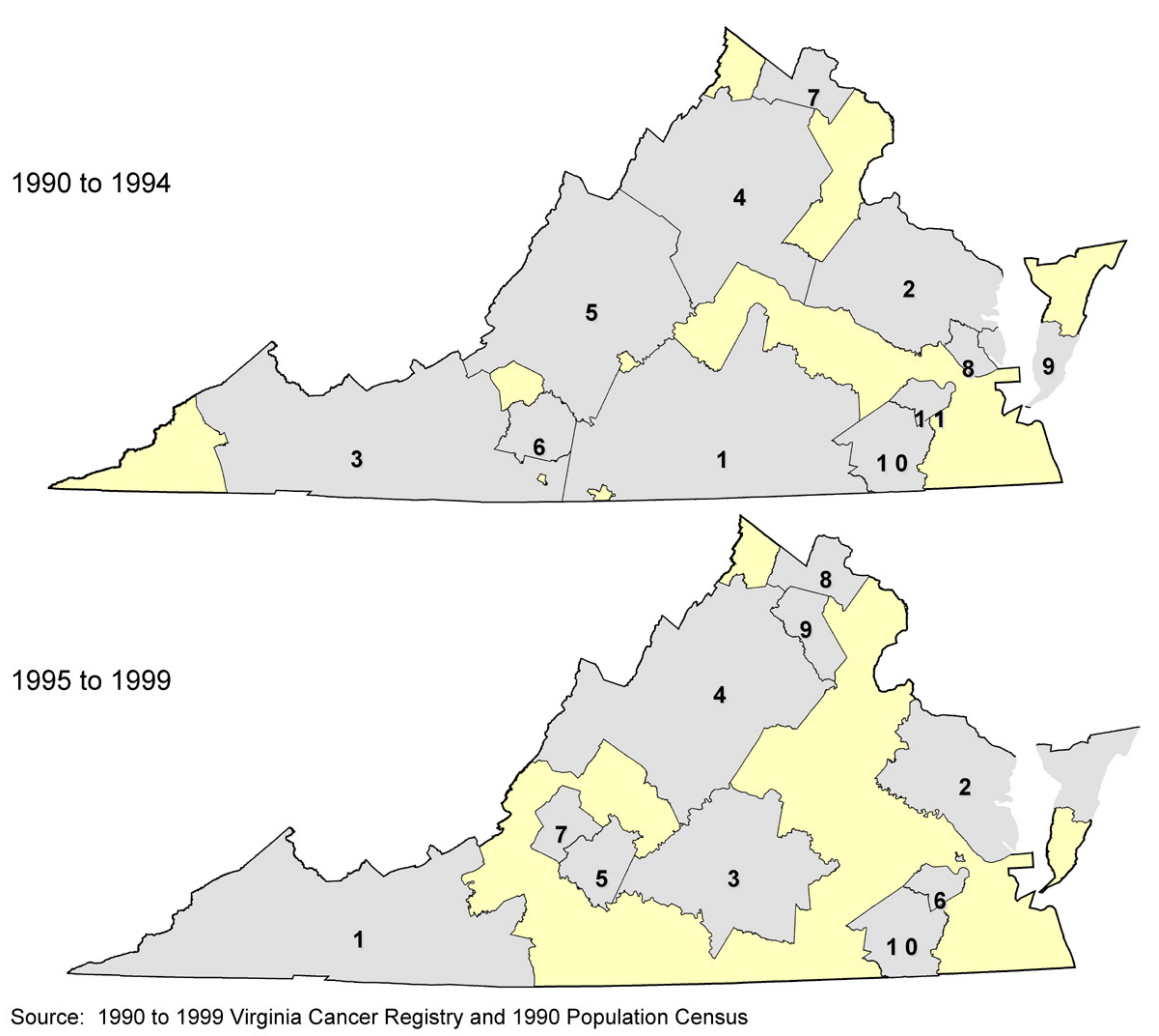
Clusters by proportion of unmatched prostate cancer cases in Virginia, 1990–99.

However, cases in the most rural portions of the state remained systematically over-represented in the group with missing geocodes. Figure 4 depicts a cluster analysis on proportion of missing geocodes. Significant clusters of missing geocodes are all in rural areas of the study area. In addition to the missing geocodes being concentrated in rural areas, the generalized linear regression analysis showed that, in the most rural counties, the percent of a county's population age 65 or older and adults with less than a high school education were both independently associated with a higher percent of missing geocodes (p = 0.016 and p = 0.003, respectively). One study in California suggested that P.O. Box holders were not necessarily representative of the entire case population [27]. Semivariograms of the residuals from the generalized linear regression models showed no sign of spatial correlation.

## Discussion

One of the most important contributions of using GIS technology in epidemiologic research is to help us discern geographic patterns of disease. We found that geographic patterns of prostate cancer incidence at the census-tract level in Virginia may reflect the distribution of the available data rather than real, underlying disease patterns. In an analysis of cancer incidence, where the census population is the denominator, information bias may result from missing geocoded data. The effect of missing geocoded data could be different, for example, in an analysis of the proportion of late-stage disease, where the denominator would be the successfully geocoded cases. However, even in this situation, if screening practices are different in urban versus rural communities, then the apparent proportion of late-stage disease could be biased.

The percentage of data able to be geocoded at the census-tract level in this analysis increased over the study period, reflecting the progressive implementation of the enhanced 911 rules by the Federal Communications Commission. These rules, among other things, require assigning street addresses to rural locations. Despite these improvements, as well as increasing prostate cancer screening from 1990–94 to 1995–99, the location of high-rate clusters did not differ markedly between these two time periods. However, for each time period the spatial location of clusters among cases geocoded to the census tract versus those located in the county were vastly different.

These findings demonstrate statistically significant pattern differences resulting from spatially non-random differences in geocoding completeness across Virginia. In classic epidemiologic terms, a measure of the effect of one factor on disease risk can be biased because of its association with another factor (confounder) and the disease. *Similarly, when the factor of interest is geographic, a factor related to the disease that is not distributed randomly across the study area can confound the appearance of maps of that disease. *Appropriate interpretation of maps, therefore, requires an understanding of this phenomenon, which we call "cartographic confounding."

In this study, systematically missing data are a result of location; however, a location's urban or rural status and associated sociodemographic characteristics were found to be associated with the likelihood of missing data from that location, as well as to the likelihood of disease in that area. Spatial patterns of disease incidence, therefore, may confound cartographically the location and sociodemographic risk factors for the disease. In our study of prostate cancer incidence in Virginia, the findings that area-level measures of income and urban status are associated with increased incidence are tempered by the possibility of cartographic confounding. This problem is particularly vexing when evaluating geographic health disparities, as the possible bias of one's statistical analyses depends upon the proportion of rural population in the study [28].

Cartographic confounding is geographically based, i.e., related to location. Methods designed to account for possibly unrepresentative data, therefore, also should account for this geographic component. One approach to dealing with this problem is to minimize missing data through imputation of geocodes [29].

## Conclusion

When conducting spatial analyses, sound analyses depend upon assessing for possible bias and cartographic confounding resulting from insufficient geocoding that leads to systematically missing data. In this way, the power of geographic information science can be more effectively brought to bear on important issues of public health and the inferences from the analyses are more likely to be correct.

## Methods

### Data sources

Incidence data are from the Virginia Cancer Registry (VCR), 1990 – 1999. There were a total of 37,373 malignant neoplasms of the prostate in that period, with 27,414 in whites, 8,252 in African Americans, and 1,707 in others. Incident cases were geocoded to the street level using ArcGIS and its StreetMap USA 2000 database.(Products of the Environmental Science Research Institute, Redlands, CA.) A point-in-polygon methodology [30,31] was used to attribute 1990 census tracts to cases. Case counts were aggregated to the census tract. All cases were assigned county codes by the VCR. County codes were checked against the address-matched geocodes as a quality control measure. County codes were incorrect in only 1% of cases.

For the study period, the North American Association of Central Cancer Registries (NAACCR) reports that the VCR has 90% case ascertainment [32]. The VCR is not given NAACCR's top ranking primarily because the number of cases ascertained by death certificate only is too high or not available, depending on the year.

Area-based measures were derived from the 1990 U.S. Census data (U.S. Bureau of the Census Summary Tape File 3A). These measures were used so that presumed exposures occurred before disease incidence. The poverty variable was a measure of the percentage of persons in a census tract below the poverty level, categorized as <10%, 10 – 19%, and ≥20%. A near-poor variable measured the percent of the tract's population between 100 and 200% of the federal poverty level. The tract's median household income was also used as a variable. A low-education variable measured the percentage of persons in a census tract 25 years or older who had less than a high-school education. A high-education variable did likewise for that percentage with at least 4 years of college. The percent of a tract's population that was rural (≤50%, 51 to <100%, and 100%) was another variable. These cutpoints were chosen based on frequencies for this measure in our data. The percent of female heads of household was another predictor variable.

For both the African-American and white populations during the study period, we used an area allocation method [19,30] to produce population averages over the study period at the tract level. (The 1990 and 2000 census tracts do not match exactly, and this method was utilized to adjust for this fact.) The 1990 and 2000 county boundaries were directly comparable. As a result, we created a direct average of populations without any manipulation. The resulting averages were annualized over the 10-year study period, and these figures were used to calculate age-adjusted incidence rates of prostate cancer by the direct method utilizing the 2000 U.S. standard million [33].

### Exploratory spatial data analysis

Annualized, age-adjusted prostate cancer incidence rates for African Americans and whites were calculated at the census tract and county levels. Owing to the low case counts at younger ages, we used three age categories (<50, 50–74, and ≥75). These incidence rates were mapped at the tract and county levels.

Low case counts, sparse populations, or both result in unstable incidence rates. We used a weighted, two-dimensional, median-based smoothing algorithm called "headbanging" to reduce this noise [34], allowing patterns to emerge from the data.

### Statistical methods

Hierarchical Poisson regression modeling, using the SAS GLIMMIX macro (SAS 2001), was performed to assess prostate cancer incidence for all census tracts and counties in Virginia by the patient's age at diagnosis and sociodemographic characteristics of the census tracts. Specifically, the number of prostate cancer cases in census tract *i *(*i *= 1, ..., 1673), age group *j *(*j *= 1, 2, 3), denoted d_*ij*_, was assumed to be distributed as a Poisson random variable, with a mean n_*ij*_λ_*ij *_where n_*ij *_is the corresponding population at risk and λ_*ij *_is the incidence rate in census tract *i *and age group *j. *We assumed a log-linear rate structure, with the county or tract intercept of the regression model conceptualized as a random effect with a spatial correlation structure to account for spatial autocorrelation in the data.

The statistical analyses were stratified by racial category. Owing to the sparseness of the VCR data in other racial categories, we only analyzed data for African Americans and whites. All main effects and two-way interactions were initially screened for significance in a logistic regression model, and a final model for the hierarchical Poisson regression was constructed using stepwise, backward variable selection. Only highly significant interaction terms (p < 0.005) were retained in the logistic model to account for the multiple comparisons inherent in the selection process [35]. The full results of this study of prostate cancer incidence in Virginia, 1990–99, is available elsewhere [22].

In the current study, we conducted analyses to assess whether prostate cancer cases clustered within the study area. As noted by Waller and Gotway [20], global clustering indicates clustering exists at some point in one's study area, whereas local clustering refers to the presence of a cluster at a specific site. We evaluated the raw count data for global clustering, using Tango's Maximum Excess Events Test (MEET) [36]. The statistical code to execute the MEET was provided to the authors in R (an open-source statistical package similar to S-Plus [Insightful Corporation]) by Prof. Toshiro Tango. We also assessed the count data with a spatial scan statistic (SaTScan) [37,38] to identify statistically significant local clusters.

The patterns of missing tract identifiers were examined by generalized linear regression models in SAS 9.1 (SAS Institute, Inc. Cary, NC) that included percent of tract population over age 64, percent of tract population aged 25 or older with less than a high school education, percent of tract population aged 25 or older with at least a college education, and the median household income in the area. These factors have been found to be associated with prostate cancer incidence [4-6,22]. Moreover, in our prior Virginia study [22], the geographic distribution of missing data and that of several of these covariates was similar, which, we hypothesized, might be the result of confounding between the two.

## Authors' contributions

MNO conceived of the study, designed and coordinated the project, performed the statistical analyses, and drafted the manuscript. KAM carried out the cluster analyses. MS performed the statistical analyses for spatial clustering. FRH helped draft the manuscript. LWP helped conceive of the study, participated in its design and coordination, and helped draft the manuscript. All authors read and approved the final manuscript.
